# The non-clinical burden of febrile seizures: a systematic review

**DOI:** 10.3389/fped.2024.1377939

**Published:** 2024-04-22

**Authors:** Maria Beatrice Marangoni, Antonio Corsello, Laura Cozzi, Carlo Agostoni, Andrea Santangelo, Gregorio Paolo Milani, Robertino Dilena

**Affiliations:** ^1^ Department of Clinical Science and Community Health, University of Milan, Milan, Italy; ^2^ Struttura Complessa Pediatria, Presidio Ospedaliero Magenta, ASST Ovest Milanese, Milan, Italy; ^3^ Fondazione IRCCS Ca’ Granda Ospedale Maggiore Policlinico, Pediatric Unit, Milan, Italy; ^4^ Department of Pediatrics, AOUP Santa Chiara Hospital, Pisa, Italy; ^5^ Department of Neurosciences, Rehabilitation, Ophthalmology, Genetics, Maternal and Child Health, University of Genoa, Genoa, Italy; ^6^ Fondazione IRCCS Ca’ Granda Ospedale Maggiore Policlinico, Neuropathophysiology Unit, Milan, Italy

**Keywords:** febrile seizures, burden, parental anxiety management, public health, caregiver education, healthcare resources

## Abstract

Febrile seizures (FS) can be frightening for parents, even though they are usually harmless. Various questionnaires have been used to assess parental reactions and awareness about FS, revealing insufficient knowledge. Studies have shown that educational interventions significantly reduce parental concerns, improve knowledge, and promote better first-aid measures. Providing clear information and emotional support to parents is important to reduce their concerns and improve FS management. Healthcare providers should give comprehensive information about FS, including the risk of recurrence, and provide clear instructions on their management. The economic impact of FS includes direct and indirect costs. Studies have shown a decrease of hospitalizations and associated costs due to improved clinical adherence to guidelines, which also reduces the inappropriate use of healthcare resources. This systematic review provides a comprehensive overview of the existing literature on parental anxiety and education about FS, as well as their economic impact, aiming at identifying areas for improvement in the management of FS and providing valuable insights for healthcare providers and policymakers to better address the non-clinical burden of this condition.

## 1 Introduction

Febrile seizures (FS) represent the most common type of childhood convulsions, typically occurring between 6 months and 5 years of age without any underlying neurological disorder (1). FS are characterized by a sudden onset of seizures associated with fever, usually triggered by respiratory or gastrointestinal infections (2). While the exact pathogenesis of FS is not fully understood, they might occur as a result of several causes, including genes encoding for ion channels, inflammatory pathways (3), environmental factors and family history (4, 5).

Although FS are generally benign, they can represent a major cause of distress in caregivers. Nevertheless, the non-clinical burden of this condition is still underestimated (6, 7). Parental anxiety and education, as well as the direct and indirect costs associated with FS, are important factors that affect the clinical management and need for a specific focus (8). The subjective burden experienced by caregivers whose child experiences FS encompasses various dimensions, including concerns about the child's health, family dynamics, and the broader implications for public health. Parental education is critical in reducing anxiety and improving management of FS, resulting in a better allocation of healthcare resources (9, 10). Interestingly, the lack of knowledge about FS, including their causes and potential complications, could enhance anxiety-related parental symptoms (11). Therefore, effective communication between healthcare providers and caregivers could be pivotal in addressing these concerns and improving parental education about FS. This review aims to analyze the multifaceted impact of FS on caregivers, exploring the subjective burden they face and its implications. Specifically, we seek to evaluate current evidence on parental stress and anxiety, factors influencing the management of FS in case of relapses, and the potential consequences of inappropriate healthcare service utilization.

In addition to the psychological toll, FS also have a financial impact on public health and family finances (7, 12–14). The direct costs associated with FS include medical expenses, such as emergency department (ED) visits, hospitalizations, exams and follow-up care (12–14). Indirect costs, such as lost wages related to time off work, can also be significant, even in cases of a self-limited and benign condition (15).

Despite an estimated prevalence of FS between 2% and 5% in Western countries and their well-known impact on families, there is a significant gap in literature concerning the non-clinical burden of the condition (1). Understanding the subjective burden experienced by caregivers is important for healthcare providers and policymakers to develop targeted interventions that address the specific needs of these individuals. Our purpose is to identify areas for improvement in the management of FS and provide insights for healthcare providers and policymakers to better address the non-clinical burden of this condition.

## 2 Methods

In this systematic review we conducted a comprehensive literature research using three databases: PubMed/Medline, Web of Science, and Cochrane. The research was conducted using the following terms: (“febrile seizures” OR “febrile convulsions”) AND (“parents” OR “survey” OR “questionnaire” OR “parental anxiety” OR “parental education” OR “costs” OR “public health” OR “economic burden”). We limited our research to articles published in English and involving pediatric patients. Our inclusion criteria included questionnaire-based studies, as well as studies that delved into different aspects of FS, including their management, their impact on parental anxiety, and their economic implications. Conversely, we excluded studies that exclusively focused on clinical aspects of FS or those that did not directly address our primary research question. Following data were extracted from each study included: first author, year, country, population, type of questionnaire, and conclusions.

Studies were screened and eligible studies were selected according to the PRISMA statement and specific inclusion criteria. To ensure a systematic review process, two independent reviewers conducted an initial screening by evaluating titles and abstracts of the identified studies. This screening was performed to caliber the relevance of each study to our research question. Subsequently, full-text articles of potentially relevant studies were subjected to a more detailed review for inclusion in our review. In cases where discrepancies or uncertainties arose during the review process, a third reviewer was consulted to facilitate discussion and reach a consensus.

## 3 Results

A total of 15 studies were included in this review (8–11, 16–26). The literature search process is reported in the Supplementary Material Figure S1. Seven surveys (46.7%) reported an insufficient parental knowledge, suggesting the need for educational interventions (8, 16, 17, 21–23, 25). Huang et al. conducted a survey about parental knowledge and concerns regarding FS in Taiwan and showed that the majority of involved caregivers had high concerns and inadequate knowledge on first-aid practices (22). For this reason, the Authors concluded that parents with FS children should be provided with more information, emotional support, and first-aid training. Educational interventions significantly reduce parental concerns about FS, improve knowledge and first-aid efficacy (9, 20). Two-hundred and nine caregivers were recruited and assigned to either an educational program or a pamphlet receiving group, and a questionnaire was used to examine parental concerns prior to and after the interventions (9). Results showed that caregivers had higher levels of concern before interventions, mainly of their children's risk of being more vulnerable in case of fever episodes, and therefore more prone to further seizures. Moreover, they were also concerned of the eventual sequelae of FS, such as brain damage, or even death. Notably, concern scores were significantly lower after educational interventions for the training group than for the pamphlet group (9).

The study conducted by Sakai et al. in 2009 compared maternal knowledge and perception of fever, fever management and information sources of mothers of children with and without a history of FS (26). The authors administered a questionnaire to mothers of children who visited health departments for a routine 18-month-old well baby check-up, collecting a total of 386 responses. They found out that mothers of children with a history of febrile seizures demonstrated a higher rate of accuracy in their knowledge of fever than those in the other group. Mothers of children with a history of febrile seizures used personal communication, whereas those in the other group relied on mass communication for health information. Providing accurate information to family members is essential to provide mothers with both accurate information and emotional support.

Another Swiss study examined parental behavior and emotional response during and after a FS episode in their children (11). Authors highlighted that FS were unknown to 44% of parents; 91% of parents reported severe anxiety on witnessing the first episode; 60% of parents thought their child would have died. Interestingly, severe anxiety was more frequently experienced by parents with lack of knowledge about the overmentioned condition and lower educational level. Moreover, 72% of parents did not know how to handle the first FS of their child, thus often leading to inappropriate measures.

A survey conducted in South Korea assessed the level of anxiety in mothers of children with FS and aimed at identifying factors associated with higher levels of concern (24). The study showed that major factors associated with maternal anxiety were uncertainty, frequency of FS, income, and information about FS. Another study confirmed that the major factor directly associated with parental anxiety is lack of knowledge regarding the management of seizures (18).

A Japanese study investigated parental thoughts and actions when their child experienced the first FS and medical management before reaching the hospital (25). The study found that parents without prior knowledge were more likely to think that FS were life-threatening; they also managed the convulsion less appropriately than parents with prior knowledge. A similar study investigated awareness, concerns, attitudes of mothers of children presenting a first episode of FS and found out that the majority of mothers did not recognize the seizure, and notably 39% of them interpreted the seizures as death; the main concern among parents was the state of their child's health in the future, followed by the fear of recurrence (23). While 76% of mothers lacked awareness regarding preventive measures, 68% demonstrated an acceptable understanding of the necessary steps in the event of a recurrence. Mothers with a higher educational level exhibited greater awareness of preventive measures (23). Analogue conclusions were reached by Rice et al., who found how only one-third of parents realized that their child had a FS when the event occurred, and most of them expressed extremely high levels of anxiety during the first episode (8).

A recent consensus involving child neurologists and pediatricians from five European countries was used to establish a consensus on the key messages to share with parents following a FS (27). Although there were some differences in emphasis between the two groups of experts, both converged on the main key messages to share with parents. This included reassurance based on epidemiology, underlying mechanisms, and emergency management, as well as information about risk factors and differential diagnoses. Importantly, both groups of experts highlighted the risks associated with medication overuse. They stated that parental information would be effective if it improved understanding, decreased parental anxiety and post-traumatic stress disorder, and prevented the modification of usual parenting behaviors.

Table 1 presents the findings from the included studies, encompassing surveys on various aspects of parental education. The data highlights a general lack of knowledge regarding FS among parents of children and toddlers.

**Table 1:** Included studies on parental reactions to FS.

First author, year, country	Population	Methods	Conclusions
Rutter et al. 1978, UK (17)	89 parents completed a questionnaire survey at the arrival at the hospital	Questionnaire on medical and parental management of children having their first FS and carried to the ED	Parental management of the first episode of FS is often inappropriate. Educational interventions are needed to improve FS management
Shuper et al. 1996, Israel (18)	46 questionnaires directed to parents after their child had the first simple FS	Questionnaire on factors associated with parental anxiety and its relief during and after 2 weeks from admission of their child	Parental anxiety related to FS is mainly due to lack of knowledge regarding the management of seizures. Parental instruction is insufficient in handling the child in case of recurrence
Huang et al. 1998, Taiwan (19)	129 parents in a case control study underwent an educational program and completed a six-part structured questionnaire	Questionnaire to parents who took their children to eleven clinics. Parents were assigned to experimental or control group. The experimental group was trained in a 2-h educational program	An educational program can effectively reduce concerns, improve knowledge, and promote adequate first aid measures among parents of FS children
Van Stuijvenberg et al. 1999, Norway (20)	181 parents responded to a questionnaire while they participated in a randomized controlled trial of ibuprofen to prevent FS	Questionnaire on parents’ perceptions and knowledge about FS, whole their children participated in a randomized controlled trial of ibuprofen to prevent FS	Parental fear of FS is a major problem with several negative consequences for daily family life. Providing adequate information may reduce parental anxiety
Ling et al. 2000, Malaysia (21)	58 parents completed a questionnaire survey	Descriptive study conducted over a 4-month period on parents admitted to pediatric wards with a questionnaire on parental response towards FS and their understanding of home management of FS conducting at the day of discharge or on the third day (if admission of more than 3 days)	Most parents who witness the first episode of FS in their child are frightened; they must be correctly educated to improve home management
Flury et al. 2001, Switzerland (11)	135 questionnaires on parents’ behavior and emotional situation during and after an episode of FS	Questionnaire on parents’ behavior and emotional situation during and after a pediatric FS, was sent to parents of children hospitalized at least 24 h	Knowledge about FS among parents is still low and providing exhaustive information can reduce parental anxiety level and lead to appropriate actions in case of FS recurrence
Huang et al. 2001, Taiwan (9)	209 parents were recruited and assigned into program or pamphlet groups by their choices	A questionnaire used to examine parental concerns in parents from southern Taiwan assigned into program or pamphlet groups by their choices	Parental concern scores about FS were significantly lower after educational interventions than prior to them
Parmar et al. 2001, India (16)	140 parents of consecutive children admitted in hospital with FS	Questionnaire on parental knowledge and fears about FS, conducted in a tertiary care center over a period of 1 year	The great majority of FS children's parents experience a high level of anxiety related both to the episode itself and to long-term consequences. Better information is required to reduce parental concerns
Huang et al. 2002, Taiwan (22)	326 parents were mailed with a questionnaire from 11 EDs	Questionnaire mailed to parents	Most FS parents have inadequate knowledge, high anxiety levels and improper first-aid practices, so they need information, emotional support, and first-aid demonstrations
Huang et al. 2006, Taiwan (10)	216 parents completed the test, and 64 completed the questionnaire again 2 weeks later	Questionnaire created via literature review, interview and expert consultation for parents visiting ED with a child with initial FC in the previous 6 months	For research purposes, the KACP questionnaire is moderately reliable to measure parental responses. Cross-cultural investigation of the questionnaire is needed to promote its use in other countries
Kolahi et al. 2009, Iran (23)	126 mothers of consecutive children presenting with FS	Interview for mothers of children with CF conducted in the second day of admission in the hospital	Parental knowledge about FS is poor and it affects the efficacy of first-aid measures
Ju et al. 2011, South Korea (24)	102 mothers whose children had a FS in five general hospitals	Questionnaire on anxiety and associated factors in mothers of children with FS admitted in five general hospitals	The most significant predictor of anxiety among mothers of children with FS is uncertainty, so healthcare providers should provide adequate information to reduce maternal concerns about FS
Kanemura et al. 2013, Japan (25)	78 parents of children with a first FS evaluated their thoughts and actions	Questionnaire on parental knowledge, management, and fears about their child's first FS. Enrolment occurred over a 48-month period using a face-to-face or telephone interview method	Parents without prior knowledge of FS have a higher rate of thinking that FS were harmful than parents with prior knowledge and managed the convulsions less appropriately. For this reason, it is important to organize parental support groups and effective educational intervention programs
Rice et al. 2022, Germany (8)	65 parents of children with a first episode of FS with a control group of 54 parents	Prospective observational study with a questionnaire on parental knowledge, management, and fears about their child's first FS	32% of parents said they knew their child had an FS when the first event occurred, and 89% described fear when the child had a seizure, with a median intensity of 10/10. Information about FS and their management should be more available to improve parents’ coping and patient safety
Sakai et al. 2009, Japan (26)	386 mothers of children who visited check-up	A questionnaire was used to survey mothers of children who visited one of the five health departments in Tokyo for a routine 18-month-old well baby check-up during February and March 2006	Mothers of children with a history of febrile seizures demonstrated a higher rate of accuracy in their knowledge of fever than those in the other group. Mothers of children with a history of febrile seizures used personal communication, whereas those in the other group relied on mass communication for health information. Providing accurate information to family members is essential to provide mothers with both accurate information and emotional support

## 4 Discussion

Although the occurrence of FS in children is frequent and mostly benign, it can be frightening and perceived as life-threatening by parents who witness them (7). Main immediate fears are about death and cerebral damage, although they are extremely rare events (28). Few cases of death following FS have been described in literature in past years, mainly attributed to neuronal necrosis due to systemic anoxia (29, 30). At the current state of knowledge, studies demonstrated that simple FS do not cause neurological damage, whereas complex ones may be associated with cerebral lesions at neuroimaging (31). When a child experiences a first episode of FS, parents may experience a range of reactions, encompassing both physical and psychological responses (7). Physically, they may grapple with symptoms such as a loss of appetite, sleep disturbances, including insomnia, and gastrointestinal discomfort, which can, in some instances, even lead to weight loss (16, 32).

**Figure 1:**
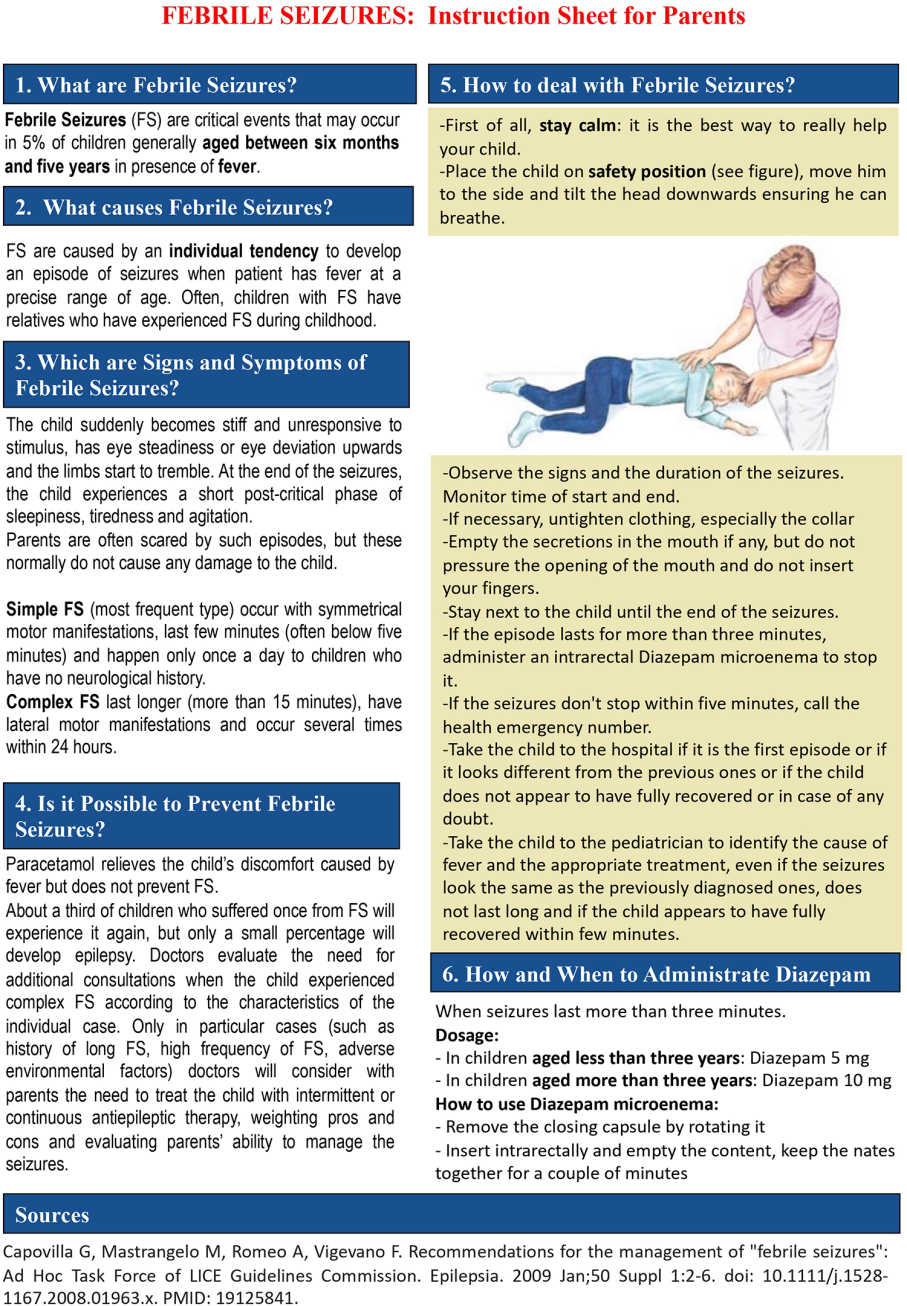
A sample of the information leaflet that should be given to caregivers by health care providers.

The studies included into this review suggest that providing clear information and emotional support to parents is important to reduce their concerns, improve FS management and adopt appropriate measures in case of recurrence. Since uncertainty is often reported as the main predictor factor of parental anxiety, doctors should give exhaustive information about FS to reduce it. Parents should be informed about the features of FS and the risk of recurrence, which is estimated around 30%–50% (4, 33), varying based on the number of risk factors. They should also receive clear indications on their management, possibly with adequate information leaflets that may be delivered directly to caregivers by health care providers (Figure 1).

Psychological reactions, on the other hand, can manifest immediately or persist as delayed concerns. Immediate responses include a visceral fear of their child's mortality and apprehension about potential brain damage. In the long term, parents may wrestle with anxieties regarding the development of epilepsy, which in turn carries an inherent risk of developing disorders such as post-traumatic stress disorder, an excessive anxiety with even minor fevers, and haunting apprehensions about seizure recurrence (34). Remarkably, this emotional turmoil persists even among parents who have previously witnessed FS episodes, affecting their mental well-being (7). Following a FS episode, many parents tend to perceive their child as more fragile than their peers, a phenomenon often referred to as the “vulnerable child syndrome”. This theory, developed by Green and Solnit, implies that after a child has recovered from a potentially fatal medical condition, there is a marked difference between his normal health and parents' perception of his vulnerability; this mismatch not only heightens parental anxiety during their child's feverish episodes, but may also cast a shadow over the overall quality of family life (35).

Furthermore, this perceived vulnerability sometimes compels parents to seek medical attention more frequently, potentially increasing healthcare costs, although this connection has yet to be conclusively demonstrated.

The economic burden of FS is constituted by direct and indirect costs. Direct charges are related to visits in the ED and days of hospitalization, while indirect costs are mainly related to missed school days by children and missed days of work by parents (12, 14). Gabutti et al. estimated that 70,610 children were hospitalized for FS in the years 2006–2010 in Italy, equal to 171 cases per 100,000 inhabitants (14). As far as the economic impact of FS on public health is concerned, we distinguish two main aspects. The first issue are costs directly related to parenteral management of FS, such as the inappropriate access to the ER, the increased level of medical attention seeking supposed to follow a first FS episode (often referred to as “the vulnerable child syndrome”) and the increased level of care due to delayed treatment out of hospital. The second aspect is exclusively related to the healthcare personnel, especially to his adherence to international guidelines. In conclusion, charges of FS may be reduced by improving their management according to existing guidelines and, on the other hand, by providing parents with correct and exhaustive information on how to handle these episodes (36).

Up to date, few data are available regarding healthcare services utilization and treatment costs of children experiencing FS (7). Most studies analyzing such aspects have been in children already diagnosed with epilepsy (37, 38). A single-center study conducted in the USA evaluated the direct costs of seizures exclusively managed in the emergency room, that did not need hospitalization (12). Authors enlightened that rescue medications, administered before medical intervention by parents could effectively manage recurrent or prolonged seizures in patients who had already been diagnosed with FS or epilepsy. This evidence requires caregivers to be properly educated in assessing when medications are necessary and when medical examination is needed. The use of rescue medications, such as diazepam rectal gel or other medications (intranasal/intramuscular diazepam, and intranasal/buccal midazolam, more recently) has proven to be cost-saving and to reduce the access to emergency services (12, 39).

Huang et al. calculated the rate and the costs of hospitalization due to simple FS in patients aged less than 6 years from 2003 to 2012 in the USA (40). Authors noticed that simple FS-associated hospitalizations and the related economic costs decreased throughout the years, probably due to improved clinical adherence to guidelines set forth by AAP in 2011 (36). The use of diagnostic procedures such as neuroimaging (except for MRI), EEG and lumbar puncture decreased as well from 2003 to 2012, showing a satisfying adherence to AAP Guidelines. These guidelines emphasize the importance of finding the etiology of the febrile episode, rather than investigating the seizure itself; they do not recommend routine use of EEG and neuroimaging in neurologically healthy children. Furthermore, in most cases no intervention is needed since FS are usually brief and self-limited events. These data are consistent with the hypothesis that AAP guidelines published in 2011 reduced the improper use of healthcare resources in the management of FS.

To strengthen this theory, Chin et al. retrospectively analyzed hospital discharge records in Scotland for a first febrile or afebrile seizure in children aged 1 month to 4 years, and the subsequent prescription of rescue anti-seizure medications (ASMs). Interestingly, the study showed that adherence to UK Guidelines reduced the inappropriate use of healthcare resources (13). Authors also enlightened that there was not an over-prescription of ASMs, as recommended by the overmentioned guidelines (13). Notably, the UK Guidelines, differently from the current clinical practice in most centers in Italy, do not recommend prescribing rescue ASMs after the first episodes of FS or for single afebrile episodes (41).

According to a case-control study, children with FS do not consume excessive healthcare resources, since they have nearly identical rates of hospitalization and physician visits as the control group (42). This study observed that children who presented to the ED with a first FS did not have more medical contacts or hospitalizations during the next 6 to 7.5 years, compared with age-matched controls who had visited the same ED at the same time. Interestingly, a significant reduction in the use of pediatricians' services following the first FS episode was observed. Authors supposed that families may require consultative and emergency care services less often after going through a perceived life-threatening event.

Surprisingly, according to this study the experience of a FS had very little effect on subsequent healthcare utilization, in contrast to the “vulnerable child syndrome” theory mentioned above. According to this theory, after a child has recovered from a potentially fatal medical event, there is a mismatch between his normal health and parents' perception of his vulnerability, which can lead to increased use of health care resources. In this study, even if children with FS were initially perceived as vulnerable, their families did not seek excessive medical attention after the acute event (42). The habits encountered in family with FS could depend greatly on the specific features of the population studies and probably need to be replicated in different settings around the world. A secondary analysis of the same data was conducted to determine which children with an initial FS required excessive subsequent physician visits (43). Authors discovered that children with a known family history of febrile or afebrile seizures had fewer medical visits than those with negative family history.

A retrospective study evaluated the adherence of ED physicians to AAP Guidelines on FS in the USA in the period 2002–2003, with the objective of minimizing invasive interventions, assuming that FS are in most cases a benign event with an excellent prognosis (44). This review noted that the use of lumbar puncture in the differential diagnosis of FS is uncommon, and most patients are discharged at home instead. However, the relatively frequent use of brain CT is inconsistent with these practice guidelines, and we then probably assume an excessive consumption of healthcare resources for this practice. This study does not specifically address direct and indirect costs of FS, but it is likely that a strict adherence to AAP recommendations reduces treatment costs and service utilization.

This review emphasizes the importance of healthcare providers and policymakers addressing the non-clinical burden of FS, which are one of the most common reasons for medical seeking in pediatric EDs. Recent studies indicate that economic impact of FS is notable, although it is decreasing due to the better adherence to published guidelines. Despite recent guidelines, there is still heterogeneity in clinical management and inadequate support for parental concerns. Parents can experience different reactions to their child's FS (both physical and psychological) and are likely to consider their child more vulnerable than the peers, thus potentially increasing the use of medical resources. Nevertheless, the impact of these aspects seems extremely heterogeneous among the different studied populations. Nonetheless, effective communication and information provision to caregivers seems to be effective in mitigating the burden associated with FS. While various approaches exist, the optimal method for disseminating information to caregivers warrants careful consideration. A comprehensive approach that combines written materials, such as informative brochures, with personalized discharge interviews may enhance caregiver understanding and confidence in managing FS. Providing caregivers with written materials allows them to reference essential information at their convenience, while personalized discharge interviews offer an opportunity for healthcare providers to address specific concerns and reinforce key messages, in order to alleviate stress and anxiety.

Our findings show a significant knowledge gap about FS among parents, highlighting the need for educational interventions. Such interventions have demonstrated the potential to relieve parental anxiety, enhance knowledge and improve first-aid measures. A comprehensive parental education approach should encompass information about FS features, risk of recurrence, and proper management of future episodes. Addressing these issues can improve overall management of FS, alleviate parental anxiety, and optimize healthcare resource utilization.

## Data Availability

The original contributions presented in the study are included in the article/Supplementary Material, further inquiries can be directed to the corresponding author.
