# Supplementary figures and images for: The non-clinical burden of febrile seizures: a systematic review
Source: Front Pediatr. 2024 Apr 22;12:1377939. doi: 10.3389/fped.2024.1377939 (PMC11070526; doi:10.3389/fped.2024.1377939)

## Supplementary Material

### 1. Supplementary Figures

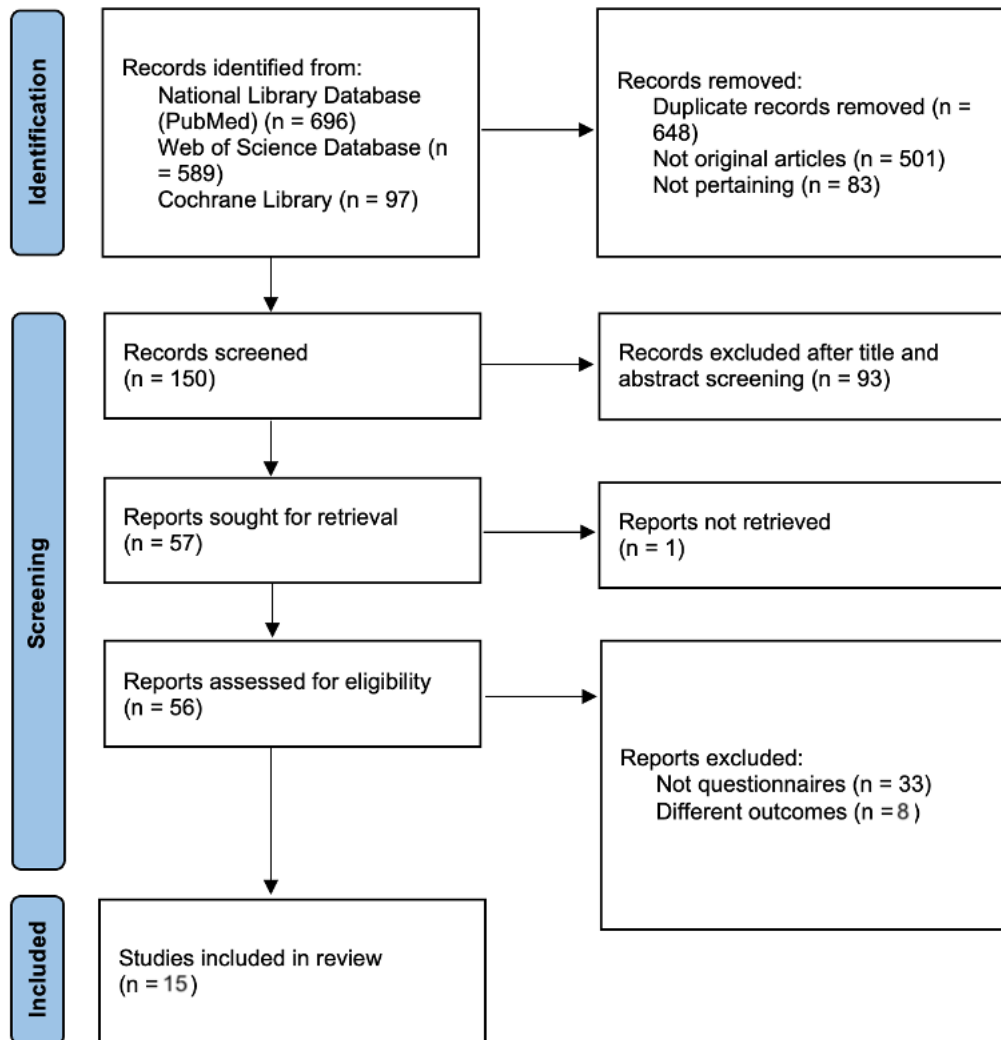

**Supplementary Figure 1.** PRISMA flow diagram.

Supplement: Supplementary file 1 [file Image1.pdf]
